# Correlation between serum leptin level and sleep monitoring indexes in patients with obstructive sleep apnea hypopnea syndrome and its predictive value: a cross-sectional analysis

**DOI:** 10.3389/fmed.2024.1346195

**Published:** 2024-04-22

**Authors:** Ji Li, Kejing Zhou, Xing Chen, Xu Lu, Deqiu Kong

**Affiliations:** ^1^Department of Otolaryngology, Head and Neck Surgery, The First Affiliated Hospital of Ningbo University, Ningbo, Zhejiang, China; ^2^Department of Ophthalmology, Ningbo Yinzhou No.2 Hospital, Ningbo, Zhejiang, China

**Keywords:** obstructive sleep apnea hypopnea syndrome, leptin, polysomnography, micro-arousal, oxygen saturation

## Abstract

**Objective:**

To investigate the association between serum leptin (LP) level and polysomnography (PSG) parameters in patients with obstructive sleep apnea hypopnea syndrome (OSAHS).

**Methods:**

A cross-sectional study was conducted. The data of subjects who underwent PSG at hospital between January 2021 and December 2022 were collected retrospectively, 220 participants were included. The subjects were categorized into simple snoring group (*n* = 45), mild OSAHS group (*n* = 63), moderate OSAHS group (*n* = 52), and severe OSAHS group (*n* = 60). The general characteristics, PSG indices, and serological indices were collected retrospectively. Pearson correlation analysis was used to observe the correlation between serum LP level and PSG parameters. The value of serum LP level in predicting OSAHS was analyzed by receiver operating characteristic curve.

**Results:**

The serum LP level was positively correlated with micro-arousal count, micro-arousal index (MAI), high apnea hypopnea index, times of blood oxygen decreased by≥3% and time in saturation lower 90%, and negatively correlated with lowest nocturnal oxygen saturation and mean oxygen saturation (*p* < 0.05). The area under the curve (AUC) of serum LP level in predicting the occurrence of OSAHS was 0.8276 (95% CI: 0.7713–0.8839), and when the Youden index was 0.587, the sensitivity was 72.00%, and the specificity was 86.67% (*p* < 0.0001). In the population with high MAI, the AUC of serum LP level in predicting the occurrence of OSAHS was 0.8825 (95% CI: 0.7833–0.9817), and when the Youden index was 0.690, the sensitivity was 79.00% and the specificity was 90.00% (*p* < 0.0001).

**Conclusion:**

Serum LP level is associated with the severity of OSAHS. Serum LP level demonstrates a strong predictive value for the occurrence of OSAHS, particularly in population with high MAI.

## 1 Introduction

Obstructive sleep apnea hypopnea syndrome (OSAHS) is a disease characterized by disrupted sleep patterns and intermittent hypoxia during the night (1). Patients with OSAHS are at an increased risk of developing cardiovascular disease, a risk that is influenced by factors such as obesity, genetic predisposition, hypertension, diabetes, and abnormal lipid metabolism. OSAHS has emerged as a significant global health concern (2). Scholars have reported that the presence of excess fat in the upper airway contributes to an increased compliance and narrowing of the upper airway in individuals with obesity, which results in decreased neural reactivity of the upper airway during sleep and an elevated risk of airway collapse, ultimately leading to the development of OSAHS (3). Consequently, obesity emerges as a significant risk factor for OSAHS, necessitating further investigation into the impact of hormones encoded by obesity genes.

Leptin (LP) is a multifunctional protein hormone that is secreted by adipose tissue. It exerts its effects on various hypothalamic pathways by crossing the blood–brain barrier as an afferent signal. LP plays a crucial role in the regulation of hunger, appetite, satiety, energy balance, as well as glucose and lipid metabolism (4, 5). Furthermore, LP also possesses the ability to modulate the diameter of the respiratory tract and contribute to the regulation of respiratory function (6). Studies have demonstrated that a considerable number of neurons implicated in the regulation of respiration possess long subtypes of leptin receptor (LepRb), namely within the nucleus of the solitary tract (NTS) and the dorsal medial hypothalamic nucleus (7). LP assumes a crucial function in the stimulation of the sodium leak channel (NALCN), thereby inducing depolarization in NTS neurons. In the neurons containing LepRb, mice with selective deletion of NALCN have respiratory rhythm changes and central respiratory pause (8). Several researchers have examined obese leptin-deficient mice and have discovered that LP plays a role in regulating the upper respiratory tract and diaphragm muscle through LP receptors, thereby influencing respiratory drive (9). Framnes and Arble suggest that elevated LP or LP resistance observed in obese people may contribute to the development of OSAHS, and that the effects of LP may also contribute the downstream signal transduction of OSAHS (10). Meszaros and Bikov concluded that OSAHS-related hyperleptinemia was related to respiratory events, sleep time, blood oxygen saturation, etc., and LP was related to the severity of OSAHS (11).

While previous studies have highlighted the significance of LP in OSAHS, there is currently few available research on the association between LP and sleep monitoring indicators in OSAHS patients. Consequently, this study aimed to gather data from subjects who underwent polysomnography (PSG), assess the factors influencing OSAHS conditions, and analyze the correlation between serum LP levels and PSG parameters, and discussed the predictive significance of serum LP level for the occurrence of OSAHS in a specific population, with the intention of offering insights for the prevention and treatment of OSAHS.

## 2 Data and methods

### 2.1 Research object

**Figure 1:**
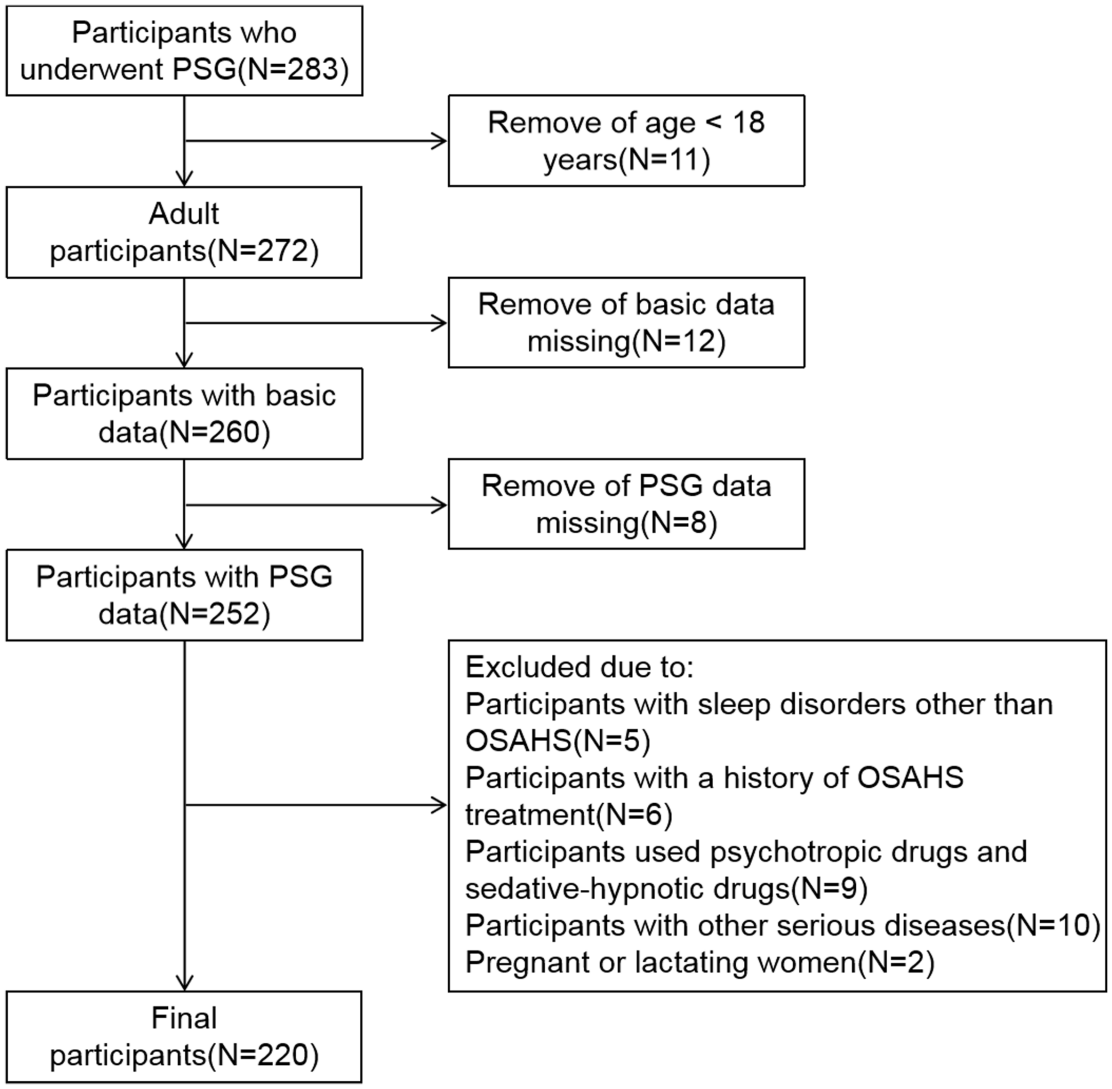
Flowchart for choosing participants.

This is a cross-sectional study, the data of subjects who underwent PSG at hospital between January 2021 and December 2022 were collected retrospectively. First, participants under the age of 18 were excluded. Subsequently, participants with missing basic data and PSG data were excluded. In addition, the exclusion criteria for all subjects are: participants with sleep disorders other than OSAHS; participants with a history of OSAHS treatment; participants used psychotropic drugs and sedative-hypnotic drugs; participants with other serious diseases; Pregnant or lactating women. Ultimately, 220 participants who met the criteria were included in the study (Figure 1). According to the apnea hypopnea index (AHI), the subjects were categorized into four groups: simple snoring (SS) group (AHI < 5 times/h, *n* = 45), mild OSAHS group (5 ≤ AHI < 15 times/h, *n* = 63), moderate OSAHS group (15 ≤ AHI ≤ 30 times/h, *n* = 52), and severe OSAHS group (AHI > 30 times/h, *n* = 60) (12). This study was approved by the Medical Ethics Committee of the First Affiliated Hospital of Ningbo University.

### 2.2 Methods

#### 2.2.1 General characteristics collection

General characteristics such as sex, age, body mass index (BMI), smoking history and drinking history were collected.

#### 2.2.2 PSG indices collection

The PSG system was employed to continuously monitor the subjects’ sleep for a minimum duration of 7 h. Prior to monitoring, subjects were instructed to abstain from smoking, consuming alcohol, consuming stimulating food, and using sleep-disrupting medications.

Definition of obstructive apnea (13): There is a drop in the peak thermal sensor excursion by ≥90% of baseline, the duration of the event lasts at least 10 s, and at least 90% of the event’s duration meets the amplitude reduction criteria for apnea.

Definition of hypopnea (13): A 30% or greater drop in flow for 10 s or longer associated with ≥4% oxygen desaturation; or a ≥ 50% drop in flow for 10 s or longer associated with a ≥ 3% oxygen desaturation or an arousal.

The monitoring results were automatically analyzed by computers and corrected by sleep experts. The total sleep time (TST), sleep efficiency (SE), the proportion of rapid eye movement (REM), the average heart rate (AHR), micro-arousal count, micro-arousal index (MAI), AHI, times of blood oxygen decreased by ≥3%, the lowest oxygen saturation (L-SaO_2_), the mean oxygen saturation (M-SaO_2_), time in saturation lower 90% (TS90%) were collected.

#### 2.2.3 Serological indices collection

In the morning following PSG, a fasting peripheral venous blood sample of 5 mL was obtained from the subjects. Subsequently, the serum was collected after undergoing centrifugation and cryopreservation. The levels of serum total cholesterol (TC), triacylglycerol (TG), high-density lipoprotein cholesterol (HDL-C) and low-density lipoprotein cholesterol (LDL-C) were determined using enzyme colorimetry on an automatic biochemical analyzer. Additionally, the levels of serum LP and Orexins (Ox) were measured using enzyme-linked immunosorbent assay.

### 2.3 Statistical processing

Data analysis was performed using SPSS 26.0. The counting data was quantified by the number of cases, and the *χ*^2^-test was employed to compare between groups. Measurement data that followed a normal distribution were represented by the mean ± standard deviation (*xˉ* ± *s*), and analysis of variance was used to compare among groups. Measurement data that did not conform to a normal distribution were represented by the median (M) and quartile range (QR), denoted as M(QR). The Kruskal-Wallis test was employed to compare among groups. Pearson correlation analysis was conducted to examine the correlation between serum LP levels and PSG parameters. The predictive capability of serum LP level in determining the occurrence of OSAHS was assessed using receiver operating characteristic curve (ROC). *p* < 0.05 was statistically significant.

## 3 Results

### 3.1 Comparison of basic data between SS group and OSAHS group

In the SS group, mild OSAHS group, moderate OSAHS group and severe OSAHS group, there were statistically significant differences in micro-arousal count, MAI, AHI, times of blood oxygen decreased by ≥3%, L-SaO_2_, TS90% and LP among the four groups (*p* < 0.05). There was no significant difference in sex, age, BMI, smoking history, drinking history, TST, SE and proportion of REM, AHR, M-SaO_2_, levels of TC, TG, HDL-C, LDL-C, and Ox among the four groups (*p* > 0.05). As shown in Table 1.

**Table 1:** Comparison of basic data between SS group and OSAHS group.

Item	SS group (*n* = 45)	Mild OSAHS group (*n* = 63)	Moderate OSAHS group (*n* = 52)	Severe OSAHS group (*n* = 60)	χ^2^/*F*/*H*-value	*P*-value
Sex					1.214	0.750
Male	34(75.56%)	47(74.60%)	37(71.15%)	48(80.00%)		
Female	11(24.44%)	16(25.40%)	15(28.85%)	12(20.00%)		
Age(years)	45.47 ± 9.12	46.35 ± 9.70	46.81 ± 8.88	46.55 ± 10.57	0.173	0.914
BMI(kg/m^2^)	26.22 ± 2.77	26.26 ± 3.18	27.11 ± 3.55	27.48 ± 2.60	2.383	0.070
Smoking history					0.680	0.878
With	17(37.78%)	22(34.92%)	21(40.38%)	25(41.67%)		
Without	28(62.22%)	41(65.08%)	31(59.62%)	35(58.33%)		
Drinking history					1.548	0.671
With	16(35.56%)	25(39.68%)	23(44.23%)	28(46.67%)		
Without	29(64.44%)	38(60.32%)	29(55.77%)	32(53.33%)		
TST(min)	382.76 ± 104.80	408.54 ± 67.61	401.46 ± 24.34	377.18 ± 78.38	2.402	0.069
SE(%)	69.40 ± 14.05	73.88 ± 7.03	71.74 ± 7.68	70.39 ± 8.94	2.339	0.074
Proportion of REM	14.46 ± 5.12	14.60 ± 5.13	15.76 ± 5.42	16.74 ± 4.39	2.573	0.055
AHR(bpm)	62.51 ± 4.61	60.68 ± 4.29	62.10 ± 6.77	63.03 ± 4.70	2.334	0.075
Micro-arousal count (times)	34(23.5)	46(32)	58.5(31.75)	62.5(29.5)	41.712	<0.0001
MAI(times/h)	5.7(3.7)	7(4.9)	8.95(5.02)	9.9(6.42)	38.396	<0.0001
AHI(times/h)	0.8(1.6)	9(4.6)	24.45(5.85)	56.2(18.57)	204.678	<0.0001
Times of blood oxygen decreased by≥3%(times)	3(6.5)	51(46)	151(73.5)	274(97.5)	191.271	<0.0001
L-SaO_2_(%)	87.29 ± 4.27	84.67 ± 5.90	77.38 ± 6.54	74.25 ± 7.69	50.175	<0.0001
M-SaO_2_(%)	95.22 ± 1.35	94.52 ± 2.18	94.79 ± 0.96	94.12 ± 3.33	2.246	0.084
TS90%(min)	0.20 ± 0.33	18.14 ± 5.32	28.44 ± 9.35	96.16 ± 51.35	132.103	<0.0001
TC(mmol/L)	4.59 ± 0.39	4.64 ± 0.35	4.69 ± 0.40	4.69 ± 0.44	0.682	0.564
TG(mmol/L)	1.69 ± 0.26	1.71 ± 0.27	1.72 ± 0.28	1.75 ± 0.31	0.374	0.772
HDL-C(mmol/L)	1.36 ± 0.19	1.39 ± 0.25	1.37 ± 0.25	1.32 ± 0.26	1.038	0.377
LDL-C(mmol/L)	2.79 ± 0.44	2.80 ± 0.38	2.82 ± 0.49	2.85 ± 0.40	0.197	0.898
LP(μg/L)	7.26 ± 2.55	8.01 ± 2.35	14.56 ± 4.14	17.62 ± 4.30	117.760	<0.0001
Ox(μg/L)	382.65 ± 161.18	390.45 ± 172.03	399.32 ± 154.38	413.14 ± 161.50	0.350	0.789

SS, simple snoring; OSAHS, obstructive sleep apnea hypopnea syndrome; BMI, body mass index; TST, total sleep time; SE, sleep efficiency; REM, rapid eye movement; AHR, average heart rate; MAI, micro-arousal index; AHI, apnea hypopnea index; L-SaO_2_, the lowest oxygen saturation; M-SaO_2_, mean oxygen saturation; TS90%, time in saturation lower 90%; TC, total cholesterol; TG, triacylglycerol; HDL-C, high-density lipoprotein cholesterol; LDL-C, low-density lipoprotein cholesterol; LP, leptin; Ox, Orexins.

### 3.2 Correlation between serum LP level and clinical indexes

Pearson correlation analysis showed that the serum LP level was positively correlated with micro-arousal count, MAI, AHI, times of blood oxygen decreased by≥3% and TS90%, while the serum LP level was negatively correlated with L-SaO_2_ and M-SaO_2_ (*p* < 0.05). There was no significant correlation between serum LP level and age, BMI, TST, SE and proportion of REM, AHR, TC, TG, HDL-C, LDL-C, and Ox (*p* > 0.05). As shown in Table 2.

**Table 2:** Correlation between serum LP level and clinical indexes.

Item	LP		
	*r*-value	*R*^2^*-*value	*P*-value
Age	0.006	0.000	0.929
BMI	0.111	0.012	0.102
TST	−0.041	0.002	0.545
SE	−0.028	0.001	0.676
Proportion of REM	0.111	0.012	0.100
AHR	0.076	0.006	0.264
Micro-arousal count	0.345	0.119	<0.0001
MAI	0.333	0.111	<0.0001
AHI	0.714	0.510	<0.0001
Times of blood oxygen decreased by≥3%	0.712	0.507	<0.0001
L-SaO_2_	−0.550	0.302	<0.0001
M-SaO_2_	−0.143	0.020	0.034
TS90%	0.554	0.307	<0.0001
TC	0.079	0.006	0.245
TG	0.089	0.008	0.190
HDL-C	−0.127	0.016	0.060
LDL-C	0.078	0.006	0.250
Ox	0.058	0.003	0.395

LP, leptin; BMI, body mass index; TST, total sleep time; SE, sleep efficiency; REM, rapid eye movement; AHR, average heart rate; MAI, micro-arousal index; AHI, apnea hypopnea index; L-SaO_2_, the lowest oxygen saturation; M-SaO_2_, mean oxygen saturation; TS90%, time in saturation lower 90%; TC, total cholesterol; TG, triacylglycerol; HDL-C, high-density lipoprotein cholesterol; LDL-C, low-density lipoprotein cholesterol; Ox, Orexins.

### 3.3 The value of serum LP level in predicting the occurrence and severity of OSAHS

**Figure 2:**
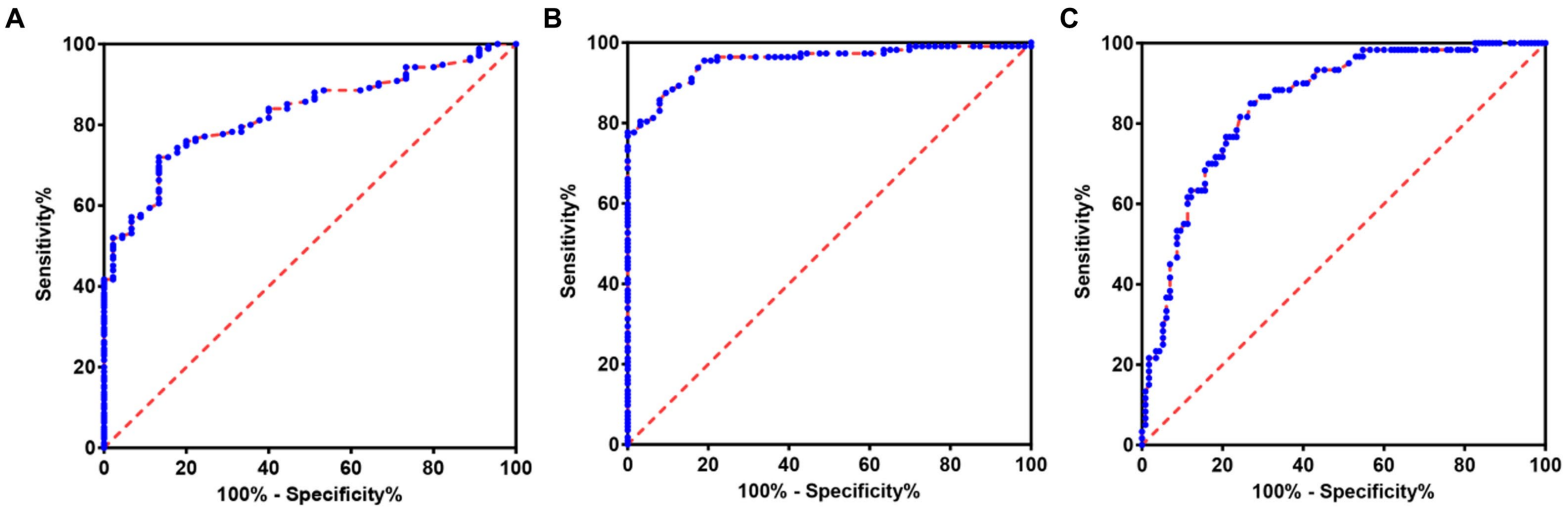
ROC of serum LP level predicting the occurrence and severity of OSAHS. **(A)** ROC of serum LP level predicting the occurrence of OSAHS. **(B)** ROC of serum LP level predicting mild OSAHS and moderate and severe OSAHS. **(C)** ROC of serum LP level predicting mild and moderate OSAHS and severe OSAHS.

In order to further explore the auxiliary diagnostic value of serum LP level in the occurrence of OSAHS, ROC curves were drawn to classify patients with OSAHS and patients with SS according to serum LP level, and searched for the best cut-off value. The results showed that the area under the curve (AUC) of serum LP level in predicting the occurrence of OSAHS was 0.8276 (*p* < 0.0001, 95% CI: 0.7713–0.8839), and the cut-off value was 9.650 μg/L (Youden index was 0.587, sensitivity was 72.00%, and specificity was 86.67%). In addition, the AUC of serum LP level in predicting mild OSAHS and moderate and severe OSAHS was 0.9548 (95%CI: 0.9256–0.9840), and when the Youden index was 0.780, the sensitivity was 87.50% and the specificity was 90.48% (*p* < 0.0001); the AUC of serum LP level in predicting mild and moderate OSAHS and severe OSAHS was 0.8508 (95%CI: 0.7935–0.9081), and when the Youden index was 0.580, the sensitivity was 85.00% and the specificity was 73.04% (*p* < 0.0001). As shown in Table 3 and Figure 2.

**Table 3:** The value of serum LP level in predicting the occurrence and severity of OSAHS.

Item		AUC	95% CI		SE	*P*	Sensitivity	Specificity	Youden index
			Lower limit	Upper limit					
LP	SS and OSAHS	0.8276	0.7713	0.8839	0.029	<0.0001	72.00%	86.67%	0.587
	Mild OSAHS and moderate and severe OSAHS	0.9548	0.9256	0.9840	0.015	<0.0001	87.50%	90.48%	0.780
	Mild and moderate OSAHS and severe OSAHS	0.8508	0.7935	0.9081	0.029	<0.0001	85.00%	73.04%	0.580

LP, leptin; SS, simple snoring; OSAHS, obstructive sleep apnea hypopnea syndrome; AUC, area under the curve.

### 3.4 The value of serum LP level in predicting the occurrence of OSAHS in a specific population

**Figure 3:**
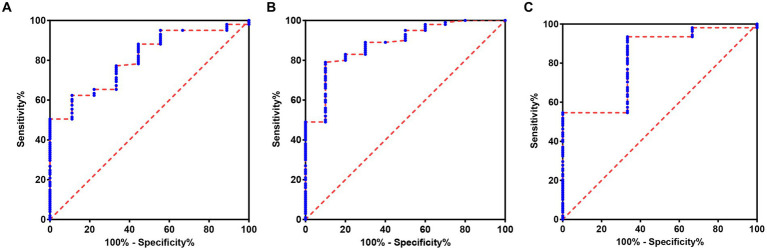
ROC of serum LP level predicting the occurrence of OSAHS in a specific population. **(A)** ROC of serum LP level predicting the occurrence of OSAHS in the population with high micro-arousal count. **(B)** ROC of serum LP level predicting the occurrence of OSAHS in the population with high MAI. **(C)** ROC of serum LP level predicting the occurrence of OSAHS in the population with low L-SaO_2_.

In order to further explore the auxiliary diagnostic value of serum LP level in the occurrence of OSAHS in a specific population, further subtype analysis was carried out on the data with differences among the four groups (micro-arousal count, MAI, AHI, times of blood oxygen decreased by≥3%, L-SaO_2_, TS90%). The median value was used as the limit for typing. Among them, different subtypes of micro-arousal count, MAI and L-SaO_2_ could be used for ROC analysis of serum LP level to predict the occurrence of OSAHS. ROC curves were drawn to classify patients with OSAHS and patients with SS according to serum LP level. In the population with high micro-arousal count (micro-arousal count >52.5 times), the AUC of serum LP level in predicting the occurrence of OSAHS was 0.8080 (95% CI: 0.6879–0.9282), and when the Youden index was 0.513, the sensitivity was 62.38% and the specificity was 88.89% (*p* = 0.002). In the population with high MAI (MAI > 7.65 times/h), the AUC of serum LP level in predicting the occurrence of OSAHS was 0.8825 (95% CI: 0.7833–0.9817), and when the Youden index was 0.690, the sensitivity was 79.00% and the specificity was 90.00% (*p* < 0.0001). In the population with low L-SaO_2_ (L-SaO_2_ ≤ 81%), the AUC of serum LP level in predicting the occurrence of OSAHS was 0.8210, but the classification effect of this model was not significant (*p* > 0.05). As shown in Table 4 and Figure 3.

**Table 4:** The value of serum LP level in predicting the occurrence of OSAHS in a specific population.

Item		AUC	95% CI		SE	*P*	Sensitivity	Specificity	Youden index
			Lower limit	Upper limit					
LP	In the population with high micro-arousal count	0.8080	0.6879	0.9282	0.061	0.002	62.38%	88.89%	0.513
	In the population with high MAI	0.8825	0.7833	0.9817	0.050	<0.0001	79.00%	90.00%	0.690
	In the population with low L-SaO_2_	0.8210	0.5945	1.047	0.115	0.058	93.52%	66.67%	0.602

LP, leptin; OSAHS, obstructive sleep apnea hypopnea syndrome; MAI, micro-arousal index; L-SaO_2_, the lowest oxygen saturation; AUC, area under the curve.

### 3.5 Typical cases

**Figure 4:**
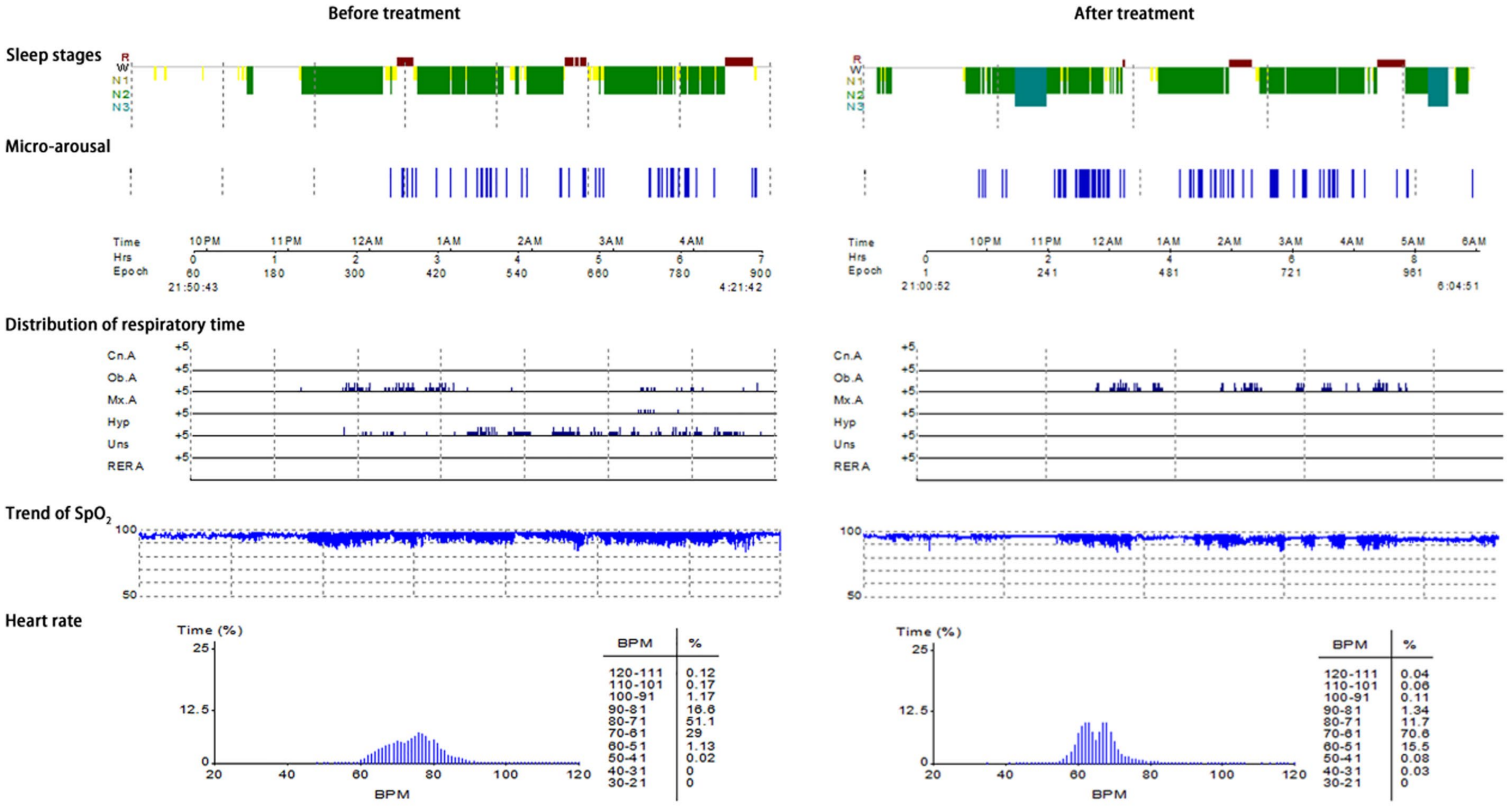
PSG monitoring report of severe OSAHS patients.

Typical case 1: A 44-year-old male patient with severe OSAHS was admitted to hospital due to symptoms including frequent snoring, apnea, wake up from suffocation at night, and daytime somnolence. The patient had a BMI of 31.7 kg/m^2^, a 10-year smoking history, and no alcohol abuse history. Sleep structure: TST of 294.5 min, sleep latency of 70.0 min, REM latency of 105.0 min, SE of 70.0%, micro-arousal count of 46 times, MAI of 9.4 times/h, sleep stage transition times of 77 times. The proportion of sleep stages: N1 stage accounted for 14.1%, N2 stage accounted for 72.0%, N3 stage accounted for 0.0%, REM stage accounted for 13.9%. No abnormal manifestations of nocturnal molars and REM phase-related disorders were observed. Respiratory events: AHI of 69.7 times/h, maximum apnea time of 52 s, maximum hypopnea time of 60s. The heart rate slowed down when respiratory events occurred, and increased after respiratory events, and accompanied by audible snoring. Blood oxygen status: M-SaO_2_ during sleep of 95%, L-SaO_2_ during sleep of 84%. Times of blood oxygen decreased by≥3% of 328 times, TS90% of 12 min 46 s. Electrocardiographic events: AHR of 73 bpm, slowest HR of 51 bpm, fastest HR of 109 bpm. Serological detection: TC of 5.3 mmol/L, TG of 2.1 mmol/L, HDL-C of 1.5 mmol/L, LDL-C of 2.7 mmol/L, LP of 11.1 μg/L, OX of 379.5 μg/L. The medical personnel implemented noninvasive positive airway pressure ventilation to the patients and advised patients to make lifestyle modifications such as weight loss, lateral sleeping, and smoking cessation. After treatment, the symptoms of the patients were better than when they were admitted to hospital. After treatment, the sleep structure was as follows: TST of 414.0 min, sleep latency of 11.5 min, REM latency of 220.0 min, SE of 76.1%, micro-arousal count of 92 times, MAI of 13.3 times/h, sleep stage transition times of 97 times. The proportion of sleep stages: N1 stage accounted for 7.7%, N2 stage accounted for 69.3%, N3 stage accounted for 11.4%, REM stage accounted for 11.6%. No abnormal manifestations of nocturnal molars and REM phase-related disorders were observed. Respiratory events: AHI of 26.1 times/h, maximum apnea time of 41 s, maximum hypopnea time of 0 s. Blood oxygen status: M-SaO_2_ during sleep of 93%, L-SaO_2_ during sleep of 86%. Times of blood oxygen decreased by≥3% of 168 times, TS90% of 12 min 14 s. Electrocardiographic events: AHR of 64 bpm, slowest HR of 45 bpm, fastest HR of 113 bpm. Serological detection: TC of 4.9 mmol/L, TG of 1.9 mmol/L, HDL-C of 1.5 mmol/L, LDL-C of 2.6 mmol/L, LP of 9.8 μg/L, OX of 359.2 μg/L. The PSG monitoring report was shown in Figure 4.

**Figure 5:**
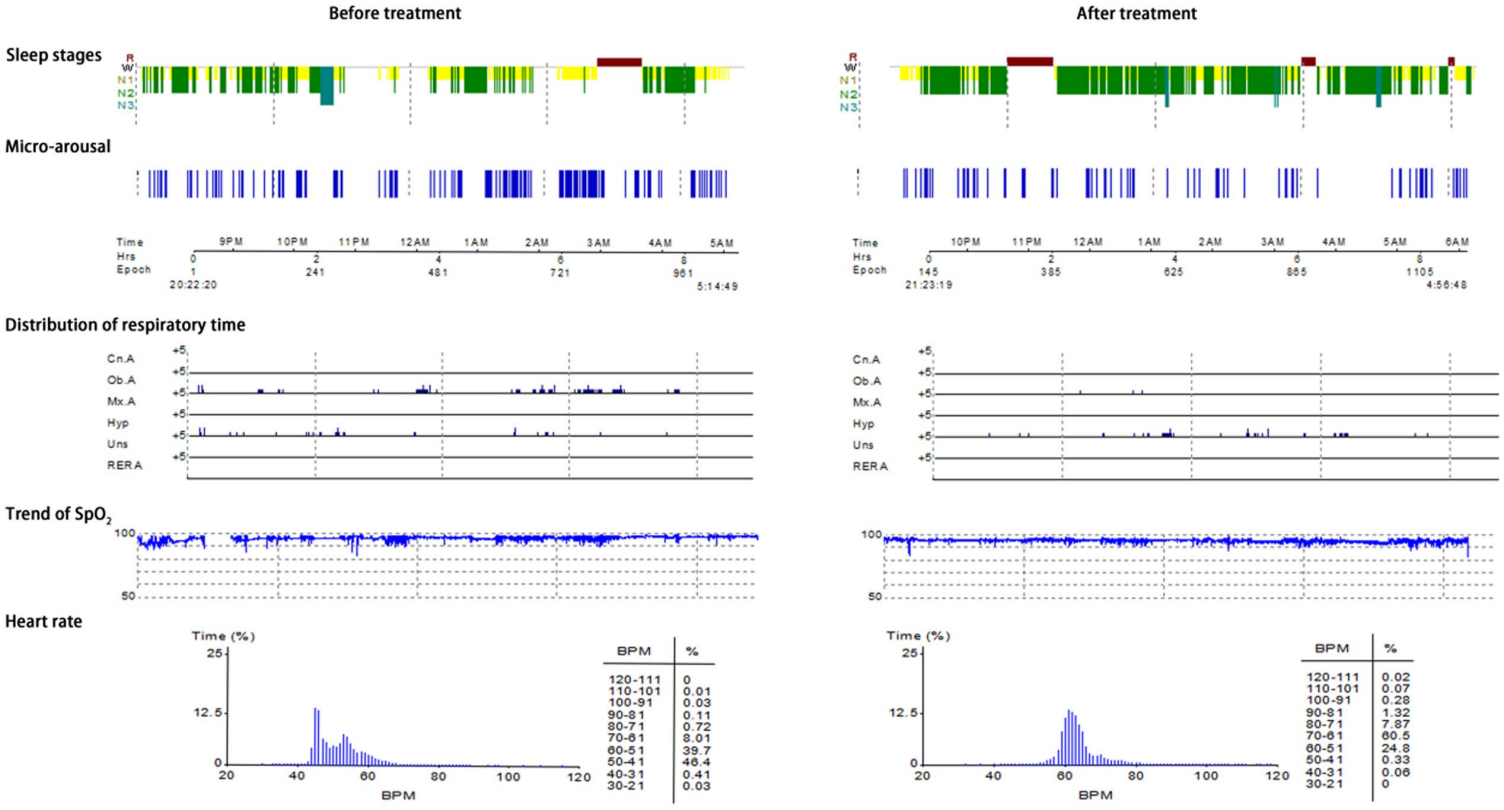
PSG monitoring report of moderate OSAHS patients.

Typical case 2: A 45-year-old male patient with moderate OSAHS was admitted to hospital due to symptoms including frequent snoring, apnea at night, and daytime somnolence. The patient had a BMI of 25.7 kg/m^2^, a 8-year smoking history, and no alcohol abuse history. Sleep structure: TST of 371 min, sleep latency of 5.0 min, REM latency of 398.5 min, SE of 67.2%, micro-arousal count of 51 times, MAI of 8.2 times/h, sleep stage transition times of 196 times. The proportion of sleep stages: N1 stage accounted for 38.7%, N2 stage accounted for 42.7%, N3 stage accounted for 3.0%, REM stage accounted for 15.6%. No abnormal manifestations of nocturnal molars and REM phase-related disorders were observed. Respiratory events: AHI of 25.5 times/h, maximum apnea time of 63 s, maximum hypopnea time of 72 s. The heart rate slowed down when respiratory events occurred, and increased after respiratory events, and accompanied by audible snoring. Blood oxygen status: M-SaO_2_ during sleep of 94%, L-SaO_2_ during sleep of 85%. Times of blood oxygen decreased by≥3% of 204 times, TS90% of 21 min 52 s. Electrocardiographic events: AHR of 55 bpm, slowest HR of 34 bpm, fastest HR of 70 bpm. Serological detection: TC of 4.2 mmol/L, TG of 1.6 mmol/L, HDL-C of 1.5 mmol/L, LDL-C of 2.4 mmol/L, LP of 13.0 μg/L, OX of 302.5 μg/L. The medical personnel implemented noninvasive positive airway pressure ventilation to the patients and advised patients to make lifestyle modifications such as lateral sleeping and smoking cessation. After treatment, the symptoms of the patients were better than when they were admitted to hospital. After treatment, the sleep structure was as follows: TST of 438.5 min, sleep latency of 33.5 min, REM latency of 86.0 min, SE of 83.5%, micro-arousal count of 78 times, MAI of 10.7 times/h, sleep stage transition times of 153 times. The proportion of sleep stages: N1 stage accounted for 22.6%, N2 stage accounted for 62.8%, N3 stage accounted for 2.2%, REM stage accounted for 12.4%. No abnormal manifestations of nocturnal molars and REM phase-related disorders were observed. Respiratory events: AHI of 7.4 times/h, maximum apnea time of 23 s, maximum hypopnea time of 43 s. Blood oxygen status: M-SaO_2_ during sleep of 94%, L-SaO_2_ during sleep of 87%. Times of blood oxygen decreased by≥3% of 105 times, TS90% of 30s. Electrocardiographic events: AHR of 62 bpm, slowest HR of 47 bpm, fastest HR of 96 bpm. Serological detection: TC of 4.0 mmol/L, TG of 1.4 mmol/L, HDL-C of 1.6 mmol/L, LDL-C of 2.3 mmol/L, LP of 11.9 μg/L, OX of 274.7 μg/L. The PSG monitoring report was shown in Figure 5.

**Figure 6:**
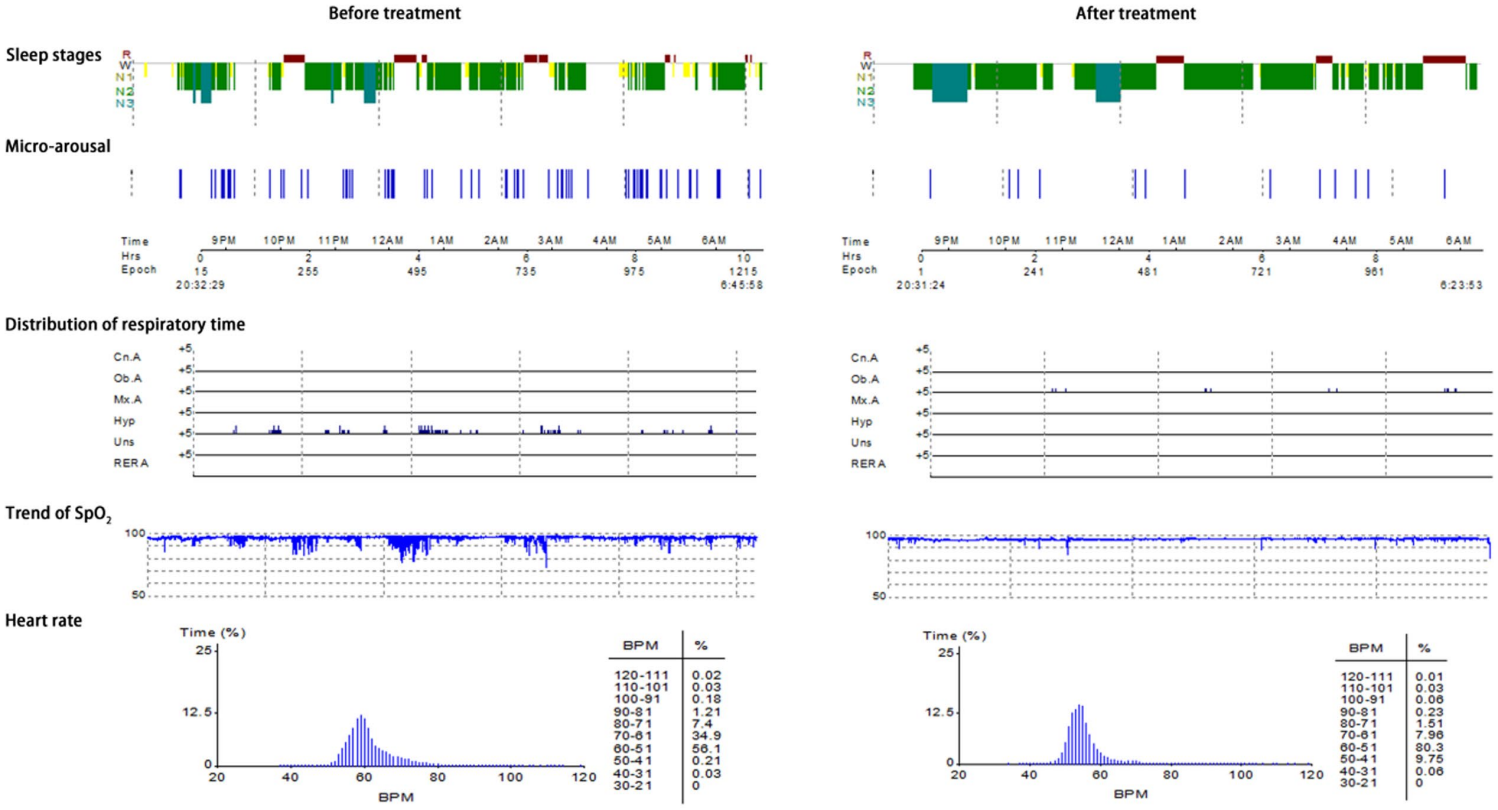
PSG monitoring report of mild OSAHS patients.

Typical case 3: A 60-year-old female patient with mild OSAHS was admitted to hospital due to symptoms including frequent snoring, apnea at night, and daytime somnolence. The patient had a BMI of 19.7 kg/m^2^, a 23-year smoking history, and no alcohol abuse history. Sleep structure: TST of 422.4 min, sleep latency of 11.5 min, REM latency of 136.5 min, SE of 71%, micro-arousal count of 70 times, MAI of 9.9 times/h, sleep stage transition times of 198 times. The proportion of sleep stages: N1 stage accounted for 16.7%, N2 stage accounted for 67.1%, N3 stage accounted for 6.2%, REM stage accounted for 10.0%. No abnormal manifestations of nocturnal molars and REM phase-related disorders were observed. Respiratory events: AHI of 5.9 times/h, maximum apnea time of 40s, maximum hypopnea time of 54 s. The heart rate slowed down when respiratory events occurred, and increased after respiratory events, and accompanied by audible snoring. Blood oxygen status: M-SaO_2_ during sleep of 93%, L-SaO_2_ during sleep of 90%. Times of blood oxygen decreased by≥3% of 67 times, TS90% of 20 min 22 s. Electrocardiographic events: AHR of 60 bpm, slowest HR of 48 bpm, fastest HR of 91 bpm. Serological detection: TC of 4.6 mmol/L, TG of 1.8 mmol/L, HDL-C of 1.3 mmol/L, LDL-C of 2.6 mmol/L, LP of 6.3 μg/L, OX of 381.7 μg/L. The medical personnel implemented noninvasive positive airway pressure ventilation to the patients and advised patients to make lifestyle modifications such as lateral sleeping and smoking cessation. After treatment, the symptoms of the patients were better than when they were admitted to hospital. After treatment, the sleep structure was as follows: TST of 505.0 min, sleep latency of 39.0 min, REM latency of 238.0 min, SE of 85.2%, micro-arousal count of 14 times, MAI of 1.7 times/h, sleep stage transition times of 54 times. The proportion of sleep stages: N1 stage accounted for 3.6%, N2 stage accounted for 68.1%, N3 stage accounted for 11.8%, REM stage accounted for 16.5%. No abnormal manifestations of nocturnal molars and REM phase-related disorders were observed. Respiratory events: AHI of 1.8 times/h, maximum apnea time of 25 s, maximum hypopnea time of 0 s. Blood oxygen status: M-SaO_2_ during sleep of 95%, L-SaO_2_ during sleep of 88%. Times of blood oxygen decreased by≥3% of 2 times, TS90% of 100 s. Electrocardiographic events: AHR of 54 bpm, slowest HR of 49 bpm, fastest HR of 78 bpm. Serological detection: TC of 3.4 mmol/L, TG of 1.2 mmol/L, HDL-C of 1.8 mmol/L, LDL-C of 2.1 mmol/L, LP of 6.0 μg/L, OX of 295.6 μg/L. The PSG monitoring report was shown in Figure 6.

## 4 Discussion

The primary symptoms of OSAHS include snoring, wake up from suffocation at night, daytime somnolence, headache in the morning, memory decline, etc. Patients may be accompanied by severe manifestations such as hypoxemia, hypercapnia and multiple organ dysfunction (14). Epidemiological data indicates that the prevalence of moderate OSAHS is approximately 50% among males and 23% among females, while the prevalence of moderate to severe OSAHS ranges from 3 to 50% (15). Studies have estimated that 936 million adults aged 30–69 years who suffer from mild and severe OSAHS worldwide, and about 425 million people suffer from moderate and severe OSAHS (16). The prevalence of obesity has risen alongside improvements in living standards, leading to an increase in the occurrence of OSAHS. The anatomical structure of the upper airway is altered due to fat accumulation in obese individuals, while the production of lipid metabolism-related hormones also contributes significantly to the development of OSAHS and obesity (17). ESADA, as a multi-center research design and a large cohort, included 11,892 patients with OSAHS. It was found that the condition of OSAHS was strongly correlated with hyperlipidemia, and severe OSAHS was more likely to occur in male, obese people and people with complications (18). However, The causal relationship between OSAHS and dyslipidemia has not been clarified in ESADA. Drager et al. conducted a meta-analysis of 13 cross-sectional studies and summarized the blood lipid status of patients with OSAHS. According to this analysis, most studies showed that compared with the control group, TC and LDL-C in OSAHS patients did not increase, and there was no significant relationship between OSAHS and lipid indexes; Some studies have found a weak association between OSAHS and blood lipids; A few studies have reported that there is a significant relationship between the severity of OSAHS and lipid concentration (19). Martínez-Cerón et al. observed 809 subjects who received PSG continuously and believed that only patients with severe OSAHS were independently correlated with dyslipidemia (20). Karkinski et al. showed that there was no statistical difference in LDL-C between OSA positive patients and OSA negative patients (21). In the authors’ study, the correlation between TC, TG, HDL-C, LDL-C and the severity of OSAHS was not found, which may be due to the influence of cohort heterogeneity and potential confounding factors on the results. There were differences in the population between authors’ study and the ESADA study, and participants’ health status, baseline BMI, trial design, and trial location may all be sources of heterogeneity. In addition, there are differences in BMI among different groups in ESADA cohort, and the prevalence rate of hyperlipidemia is also different. Compared with ESADA cohort, the subjects in the authors’ study is more likely to cause OSAHS through abnormal upper airway structure, nasal diseases, tonsil hypertrophy, etc. There is no difference in BMI among different groups of OSAHS severity, and this study was less influenced by obesity bias and had less influence on lipid levels. Therefore, there is no clear evidence to show the effect of OSAHS on dyslipidemia, and more in-depth research is still needed to observe the relationship between OSAHS and dyslipidemia.

This study found that in the SS group, mild OSAHS group, moderate OSAHS group and severe OSAHS group, there were statistically significant differences in micro-arousal count, MAI, AHI, times of blood oxygen decreased by ≥3%, L-SaO_2_, TS90% and LP among the four groups. PSG index possesses the capability to effectively differentiate between SS and OSAHS, serving as an indicator of the frequency of nocturnal respiratory events in OSAHS patients, and holds a crucial position in clinical diagnosis and application. In addition, LP is a hormone that can regulate the level and distribution of fat in the body. Its functions extend beyond promoting energy expenditure and sensitive to nutritional conditions, as it also plays a role in modulating the autonomic nervous system and endocrine system (22). Li et al. conducted a study which revealed that individuals diagnosed with OSAHS exhibited significantly elevated levels of serum LP compared to the healthy population. Furthermore, a positive correlation was observed between LP levels and the AHI (23). Studies have observed obese participants within the range of BMI > 30 to <35 kg/m^2^, and found that OSAHS patients revealed a significant prevalence of hyperleptinemia, which is related to the deterioration of OSAHS symptoms (24). The author found that serum LP level is associated with the severity of OSAHS. Moreover, serum LP level demonstrates a strong predictive value for the occurrence of OSAHS, with AUC was 0.8276, sensitivity was 72.00%, and specificity was 86.67%. In addition, the AUC of serum LP level in predicting mild OSAHS and moderate and severe OSAHS was 0.9548; the AUC of serum LP level in predicting mild and moderate OSAHS and severe OSAHS was 0.8508, which indicates that serum LP level also has application value in evaluating the severity of OSAHS. In OSAHS patients, LP exhibits a dual role, suppressing appetite while simultaneously increasing energy expenditure. However, despite the various factors contributing to hyperleptinemia in OSAHS patients, the presence of LP resistance hinders its normal physiological function and diminishes its biological effects, ultimately exacerbating fat accumulation in the upper airway and increasing respiratory resistance. Conversely, LP resistance will lead to airway obstruction and aggravate the symptoms of patients with OSAHS. In addition, in this study, the subjects’ serum LP level was positively correlated with micro-arousal count, MAI, AHI, times of blood oxygen decreased by≥3% and TS90%, while the serum LP level was negatively correlated with L-SaO_2_ and M-SaO_2_, which suggested that micro-arousal, apnea, hypoventilation and blood oxygen saturation were all factors affecting the changes of serum LP level. LP level is related to the change of arousal, which can regulate the function of neurons related to micro-arousal and increase the duration of slow-wave sleep, REM and non-REM (25). According to reports, LP increased of delta power during non-REM sleep, and induced c-Fos expression in ventrolateral preoptic nuclei and melanin concentrating hormone expressing neurons. LP act as hormonal mediators between the status of body energy and the regulation of the sleep–wake cycle (26). Patients with OSAHS commonly experience varying degrees of hypoxemia, which consequently results in sympathetic nerve excitation, increased angiotensin levels, and enhanced respiratory drive. These physiological changes may be associated with the level of LP (27). Exposure to hypoxia for 24 and 48 h can induce the expression of mediators such as LP to increase, and LP can modulate the hypoxic state of the body by positively regulating the respiratory center (28). Gauda et al. posited that an elevated level of LP stimulates the peripheral chemical reflex of hypoxia and facilitates the growth of sympathetic nerve in adipose tissue (29). There are also studies that believe that an increased percentage of rapid eye movement sleep was related to a greater reduction in LP during sleep even when controlling for age, gender, percent body fat and total sleep time, no significant relationship was observed between non-REM sleep stages and overnight change in LP levels (30). However, a study involving 73 non-obese OSAHS patients revealed that serum LP levels did not exert any influence on PSG parameters (31). The discrepancy in findings may be attributed to the fact that this research encompasses the LP levels of all BMI populations, rather than solely focusing on non-obese OSAHS patients.

It is worth noting that further subtype analysis was carried out on the data with differences among the four groups (micro-arousal count, MAI, AHI, times of blood oxygen decreased by≥3%, L-SaO_2_, TS90%), and the median value was used as the limit for typing. The results showed that in the population with high MAI, the AUC of serum LP level in predicting the occurrence of OSAHS was 0.8825 (95% CI: 0.7833–0.9817), and when the Youden index was 0.690, the sensitivity was 79.00% and the specificity was 90.00%. However, the AUC of serum LP level in predicting the occurrence of OSAHS was 0.8276, suggesting that serum LP level has a strong predictive value for OSAHS in population with high MAI, and its sensitivity and specificity are improved. Micro-arousal, defined as the awakening of EEG changes lasting for 3–15 s, holds dual importance in sleep structure. Micro-arousal may be associated with airway opening and re-establishment of airflow following respiratory events, serving as a protective mechanism; On the contrary, the occurrence of frequent micro-arousal can lead to an increase in sleep segments and sleep disorders, as well as an elevation in the frequency of respiratory events (32). The utilization of the MAI proves advantageous in number of micro-arousals per hour during sleep. A higher MAI value correlates with poorer sleep quality. The rise in MAI, influenced by chemoreceptor stimulation and awakening threshold, may amplify the risk of sleep-related complications (33). Qian et al. conducted a study involving 2,686 subjects who underwent PSG and discovered a positive correlation between MAI and the increase in LDL-C levels. This increase in MAI was found to result in sleep fragmentation, increase energy demand, affect food intake, and subsequently an elevated risk of obesity, leading to dyslipidemia (34). The author determined that the MAI of patients with OSAHS can influence the predictive effectiveness of serum LP levels for OSAHS. Consequently, in clinical, it may be advisable to consider LP detection for population with high MAI, while a comprehensive diagnosis can be achieved by combining sleep parameters and respiratory events.

To sum up, serum LP level is associated with the severity of OSAHS. Moreover, serum LP level demonstrates a strong predictive value for the occurrence of OSAHS, particularly in population with high MAI. Clinically, the occurrence and condition of OSAHS can be evaluated by combining the detection of serum LP level, clinical symptoms and sleep examination results. However, this study has some limitations. First of all, this study was a cross-sectional study, LP levels can be affected by a variety of other factors, and the interference of confounding factors such as sleep type, circadian rhythm preference, or diet structure cannot be ruled out. Then, this study lacks the treatment and follow-up data of patients with OSAHS, and fails to clarify the specific mechanism of the influence of serum LP level on sleep monitoring parameters. The author need to carry out multi-center and prospective research in the future to further confirm the conclusion of this study and reduce the one-sidedness and limitation of the results.

## Data availability statement

The original contributions presented in the study are included in the article/supplementary material, further inquiries can be directed to the corresponding author.

## Ethics statement

The study was approved by the First Affiliated Hospital of Ningbo University, in compliance with the Helsinki Declaration. The studies were conducted in accordance with the local legislation and institutional requirements. The participants provided their written informed consent to participate in this study.

## Author contributions

JL: Conceptualization, Data curation, Formal analysis, Investigation, Methodology, Writing – original draft, Writing – review & editing. KZ: Conceptualization, Data curation, Formal analysis, Project administration, Resources, Supervision, Writing – original draft, Writing – review & editing. XC: Project administration, Resources, Writing – review & editing. XL: Data curation, Writing – review & editing. DK: Supervision, Writing – review & editing.
